# Innovations to reduce demand and crowding in emergency care; a review study

**DOI:** 10.1186/s13049-014-0055-1

**Published:** 2014-09-11

**Authors:** Suzanne Mason, Gail Mountain, Janette Turner, Mubashir Arain, Eric Revue, Ellen J Weber

**Affiliations:** School of Health and Related Research, University of Sheffield, Sheffield, UK; School of Health and Related Research, Sheffield, UK; School of Health and Related Research, Sheffield, UK; Faculty of Nursing, University of Calgary, 2500 University Drive NW, Calgary, AB T2N 1 N4 Canada; Emergency Department, Louis Pasteur Hospital and Prehospital EMS, Chartres, France; Emergency Medicine, University of California, San Francisco, USA

**Keywords:** Emergency care, Emergency department, Alternative services, Patient care, Accident and emergency, Health services in England, Walk-in centres

## Abstract

Emergency Department demand continues to rise in almost all high-income countries, including those with universal coverage and a strong primary care network. Many of these countries have been experimenting with innovative methods to stem demand for acute care, while at the same time providing much needed services that can prevent Emergency Department attendance and later hospital admissions. A large proportion of patients comprise of those with minor illnesses that could potentially be seen by a health care provider in a primary care setting. The increasing number of visits to Emergency Departments not only causes delay in urgent care provision but also increases the overall cost. In the UK, the National Health Service (NHS) has made a number of efforts to strengthen primary healthcare services to increase accessibility to healthcare as well as address patients’ needs by introducing new urgent care services.

In this review, we describe efforts that have been ongoing in the UK and France for over a decade as well as specific programs to target the rising needs of emergency care in both England and France. Like many such programs, there have been successes, failures and unintended consequences. Thus, the urgent care system of other high-income countries can learn from these experiments.

## Introduction

Emergency Department (ED) visits are rising worldwide [1] and many countries are experimenting with novel methods for addressing the demand. Additionally, all health care systems are anticipating greater demand on services with the aging of the population. Despite a very strong network of general practitioners and universal health care coverage, the UK is continuing to see a rise in demand for ED services and increasingly overcrowded departments [1]. The National Health Service (NHS) is responsible for providing healthcare services in the UK and for implementing new services and healthcare related policies. Over a decade ago, the NHS and medical researchers began to address this issue by creating a variety of programs with the goal of not only reducing ED visits and costs but with the intent of more closely matching services to patient needs and preferences [2]. Before introducing a new or reconfigured healthcare service is important to assess local needs and ensure that the service is valuable and accessible to the local population. In addition, close monitoring and evaluation of new services is also required to measure impact and to maintain the quality of the services and their proper utilization by patients [3].

The issue of crowding in emergency departments has been identified in other countries and some of the measures, similar to the UK urgent care system, have been implemented. In the USA, walk in centres originated as freestanding emergency centres in the early 1970s. In late 1970s and 1980s, they evolved into ‘urgent care centres’ or ‘ambulatory care centres’. By the year 1986, around 3800 walk-in centres were dealing with 53 million patient contacts per year [4]. These centres offer lower per-episode costs than emergency departments. Urgent care centres, therefore, may reduce overall health system spending if patients substitute care at urgent care centres for care at more expensive sites [5].

This review provides examples of different interventions that have been implemented in the UK and some of the similar interventions in France to reduce avoidable demand on ED services. The evidence may provide some useful lessons and guidance for the development of urgent care systems in other developed countries.

## Methods

The Medline (Pubmed), CINHAL and Web of Science search engines were searched to identify the peer reviewed literature. In addition, Google Scholar was used for non-indexed articles and “grey” literature. This is a narrative review set out to describe the most widely used and evaluated approaches in England to manage ED demand. The innovations were identified by the authors who were experts in the area, and then the literature was searched to describe existing evidence for their impact as well as unintended consequences. Where similar efforts have been initiated in other high-income countries, these were noted as well. Most of the cited studies are UK based but studies from France were also included where relevant. Keywords used included urgent care services, walk-in centres, emergency care, accident and emergency, alternative services to emergency department, ambulatory care, minor injuries, GP out-of-hour services. As this was a narrative, scoping review no formal quality assessment was done. Most of the studies included were observational studies and surveys. Major studies were non-randomized controlled studies (before and after design and ecological studies), cross-sectional surveys and studies on secondary data (hospital records). In addition, qualitative studies were also included.

## Review

We reviewed some important interventions to reduce patient load on ED such as telephone triage, alternative primary care services, advanced nursing roles, senior doctor at the front door of the ED, emergency care in the home admission assessment units, targets for emergency departments, and interventions to manage elderly emergency care. There is mixed evidence regarding the effectiveness of these interventions; some of them are more effective than others in terms of improving patients’ access to services, and reducing patient load on EDs. There are multiple approaches to manage patient load on EDs. Approaches to manage ED load generally fall into one of the following categories: decreasing ED use, improving resources for managing the higher ED demand, improving flow into and out of the hospital to accommodate newly arriving and admitted ED patients, and elderly emergency care.

### Decreasing ED use

#### National phone advice lines

In 1998, the National Health Service of England instituted NHS Direct, a national advice line for health concerns [6]. This was a nurse led telephone service with a principal aim of providing easier and faster advice for patients. There was also an underlying expectation that it might help reduce or limit the demand on other parts of the NHS, in particular ambulance services, EDs and general practice urgent care services by providing self care advice or suggesting alternative places of care, including a routine visit to the general practitioner (GP). However, an evaluation of the early NHS Direct pilot sites conducted by the University of Sheffield showed the advice line had little impact on ambulance use or ED visits [7] and although the service subsequently became nationwide there is no evidence that it has had any impact on the demand for ED care and visits have continued to rise. More recently, NHS Direct has been replaced by a new, free to use service, NHS 111 [8]. The rationale for this change was that as options for accessing emergency and urgent care were increasing, the public were still confused about how to access services appropriately. Whilst NHS Direct was primarily an “advice and direct” service, NHS 111 is a “direct and advice” service with greater integration between the telephone triage element and other health care services. So, for example, an ambulance can be dispatched, the call transferred for additional clinical assessment or appointments with services made in a single telephone call. Unlike NHS Direct, NHS 111 uses non-clinical call handlers for the initial call assessment. The objectives of NHS 111 were to improve access by having only two telephone numbers – 999 for emergencies and 111 for urgent requests – thereby increasing public satisfaction and confidence and improving efficiency within the emergency and urgent care system by directing callers to the “right place, first time”. An expected benefit was that this would reduce avoidable ambulance journeys, ED attendances as well as emergency admissions [9]. An evaluation of the first 4 pilot sites found there was no significant impact on ED attendances and that, contrary to expectations, there was a significant increase in ambulance responses [9].

#### Alternative sites for care

In 2005 there was a national renegotiation of the General Practitioner (GP) contract allowing GPs to opt out of care of their patients on holidays, nights and weekends. The gap in service delivery was filled by a surge in the organisation of alternative sites of care and expansion of allied health professional roles. There has been a proliferation of Walk-in centres and Urgent Care Centres, generally nurse-led with some being remotely situated from EDs and others being co-located with the ED. GP-led walk-in centres were established in England in 2009 to increase GP accessibility and to decrease minor case attendance at EDs [10].

Despite the increase in the number of alternative services, studies in the UK show no significant impact of alternative services on ED attendances [11,12]. One ecological study conducted to determine the effect of walk-in centres in reducing waiting time in other primary health care services found no benefit of such centres in reducing load on other health care services [13]. Similarly, other studies reported limited evidence to support any decrease in the EM load where nurse led walk-in centres were co-located with EDs [14]. However, one study on a single walk-in centre has shown a significant decline in ED attendances as a result of the opening of the walk-in centre [15]. Patient satisfaction with quality of service is also greater in walk–in centres as a result of easy access and much shorter waiting times when compared to primary care [16]. Furthermore, the services are of standard quality and safety [17].

It is difficult to accurately measure the impact of opening a parallel service on the ED because when a new health care service is established, it creates its own demand for health care along with sharing the load of existing emergency services. Patient load on other services may actually increase if more patients are consulting these services for minor and self-limiting illnesses as a result of easy access [18]. This may lead to an increased load on health care services as well as increased referrals through these centres. There are several other reasons why alternative services may not be able to show the desired impact on ED attendances such as lack of awareness amongst the general public, patients’ perceptions about their health problems and lack of encouragement from health care providers to access alternative services [19].

The concept of walk-in centres was introduced in early 80s when the first walk in centre was opened in Canada in 1980 [20]. Canadian walk-in centres are more comparable to UK walk in centres than USA centres. Two features of Canadian centres are similar to the UK walk in centres. First, they are also funded by general taxation. Second, GPs play a gate keeper role for secondary and tertiary healthcare. A third feature which is similar to the UK is that patients are not charged for using the walk in centres which is different to the USA walk in centres [21].

Australia has recently introduced nurse led walk-in centres (2010) as a result of a shortage of GPs [22]. Australian model is based on the UK urgent care services and similar strategies have been implemented. The centres have been co-located with EDs. Consultations are provided by nurse practitioners only who get support from clinical decision support software. The aim is to complement other healthcare services and reduce patient load at other services [23]. Further evaluations need to be conducted in the future to determine the impact of these services on reducing overcrowding at EDs.

#### Emergency care in the home

Paramedic roles have extended substantially with the development in the UK of the paramedic practitioner and the emergency care practitioner. These individuals are trained to assess patients at home and can provide treatment and also referral onwards to other services when appropriate. Training aims to develop skills in history taking, examination techniques and basic investigations (such as urine testing, ECGs, referral for x-ray), and treatment options ranging from referral or signposting to other services, prescription of analgesia or antibiotics and wound management. The focus has generally been on management of minor illnesses and injury conditions, and is often aimed at the management of the frail elderly [24] and therefore incorporates an element of cognitive evaluation and social assessment. Evaluations have shown benefits to patients in reducing the need for onward journeys to the ED, high levels of patient satisfaction, high quality care with no impact on mortality and morbidity demonstrated, and reduced health service costs [25,26].

#### Technology

In common with other developed countries, UK health policy is promoting the use of technology as a means of reducing the need for face-to-face contact by health and social care, thereby controlling escalating demands upon services. A new model of health care delivery has been identified which is underpinned by information and communication technologies [27]. Telehealth is one example of such a technology and involves the remote exchange of physiological data such as blood pressure, and other vital signs between the patient and healthcare professionals who are located at a distance [28]. A variety of devices are commercially available which can be used to identify potential deterioration, prevent avoidable hospital admissions and help improve the individual’s quality of life [29]. Cited benefits of Telehealth include enabling clinicians to reprioritise their workloads and assisting people with long- term conditions to self-manage [30]. The second main form of technology, Telecare, is concerned with promoting safety and security through monitoring activity in the home and community and providing alerts when that activity falls outside anticipated parameters [31]. The UK policy push for both Telehealth and Telecare has been accompanied by a large (5830 subjects at 12 sites), randomised controlled trial of both types of technologies, which demonstrated a mixed picture on outcomes. Patients with COPD, CHF and Diabetes who received tele-monitoring (e.g. glucometer, oxygen saturation, weight monitoring) as well as motivational interventions had fewer emergency visits after adjustment for baseline characteristics and predicted risk score (IRR0.85) [32]. Further research in this area is needed to fully understand the possible benefits in terms of cost effectiveness.

### Managing demand: expanding capacity without breaking the bank

#### Advanced nursing roles

Emergency Nurse Practitioners are found among provider staff in most UK emergency departments. Whilst not reducing attendances per se, the evidence has shown that these roles can operate effectively and autonomously in managing a specific workload in the ED mainly relating to minor injury and illness, similar to their roles in many EDs in the USA [33,34]. However, some UK departments have engaged advanced practice nurses for specific streams of protocol-driven care to which patients are directly referred from their GPs, or immediately assigned on arrival to the ED. These include patients with chest pain requiring a “rule-out”, often with same-day stress testing, and evaluation and treatment for suspected deep vein thrombosis [35]. There is some concerns that these streamlined services result in more GP referrals to the ED to procure this service, but they also reduce ED waiting times, standardise care and allow predictable follow-ups without physician involvement [34].

#### Senior doctor at the front door of the ED

The involvement of senior physicians early in the patient’s visit to improve flow is increasingly occurring in the UK. The approach was first recommended by England’s Audit Commission in 1999 [36]. Various systems of senior-led initial assessment have been proposed and trialed across the UK already. The 2004 IMPACT study by Terris et al. found that senior physician initial assessment significantly reduced waiting times in the whole department, most especially for ‘minors’ patients but the effect of additional resources was not directly measured [37]. Team triage by Subash et al. also showed reduced waiting times during the intervention periods but no benefit to the ED beyond these times [38]. Other authors have also observed improved efficiency with senior physician initial assessment; however the process is not thought to be sustainable without additional staffing resources [39-41].

### Improving hospital (and therefore ED) flow

#### Admission assessment units

Most hospitals in the UK now have Medical and Surgical Assessment Units where patients admitted from the ED often go for further assessment by internists or surgeons before being admitted to a specific ward [42]. This decreases the need for extensive testing in the ED, thus reducing ED length of stay and crowding. Increasingly, the Medical Assessment Units (MAU) are staffed by “Acute Physicians,” a growing specialty in the UK*.* These are senior physicians who have no other responsibilities during their practice day, conduct twice daily patient rounds, and whose primary goal is rapid evaluation of patients. It is expected that more than 50% of patients admitted to the MAU will be evaluated and discharged within 72 hours; those who cannot be discharged are transferred to specialist wards. Because the MAU’s are physically close to the ED’s (usually on the same hospital floor), the acute physicians frequently assess patients in the ED and evaluate patients that are likely to need admission, even before the ED has called them. Finally, these units also accept direct admissions from nursing homes and GPs, allowing patients who clearly need admission and who have already been evaluated by a clinician, to bypass the ED.

A study conducted by the Royal College of Physicians in 2012 demonstrated that when these units were staffed appropriately with physicians dedicated to the units that are able to round at least twice daily, the quality of care was higher in terms of reduced case fatality ratio. In addition, a lower 28 day readmission rate was observed. However, they did not demonstrate a shorter length of hospital stay [43].

MAUs have also been established in Australia and New Zealand to reduce pressure on emergency departments [44]. There are various synonyms used by different units throughout Australia and New Zealand and which may be referred to in this document: Acute Medical Ward (AMW) or Unit (AMU), Acute Assessment Unit (AAU), Acute Medical Assessment and Planning Units (AMAPU), and Admission and Planning Unit (APU) [44].

#### Targets for emergency departments

In 2002, the UK government introduced a four-hour target, target of arrival in the ED to discharge or admission, for its emergency departments. Initially aimed at 90% of attending patients, the target quickly rose to include 98% of attending patients by 2005. A huge amount of resource and attention was poured into achieving the target, which also received a lot of media attention. Critics point out that this is merely a process measure and does little to improve quality of care. There have been further well-publicised criticisms of the standard; that it leads to ‘gaming’ and ‘cheating’ [45]. Other criticisms are that it impairs training and recruitment, reduces professional satisfaction creates an adversarial culture between the emergency department and other inpatient specialties and results in unreliable data. One study found that real-time training opportunities were being lost due to the pressures of the target, and that key skills were not being practiced because they were being referred on to other specialties for management such as complex suturing [46]. However, the pressure that the target brings cannot be convincingly and solely linked to the current recruitment difficulties in emergency medicine [46]. The target was reduced to 95% of patients in June of 2010 after the coalition government was elected. In April of 2011, the target was de-emphasised as it became one of the several indicators in a quality dashboard. Evidence has shown that the target was achievable without compromising on patient care; however, overall performance on the four-hour target has markedly deteriorated since its de-emphasis [31,46,47].

Australia has also been one of the few other countries to embrace the four-hour target concept. However, the Australian picture is more positive, with the introduction of the National Emergency Access Target being associated with a substantial decrease in mortality from 1.12% to 0.98% [48]. Similarly, Canada also introduced four-hour emergency department targets in for type V and type VI patients [49].

### Advancement in elderly emergency care

The estimates of recent increase in emergency department visits by elderly patients range from 3-6% annually in the USA, UK, Canada and Australia [50]. Elderly patients have longer length of hospital stays, more complications, and more readmissions [43]. Several programs in the UK are aimed at providing emergency care for the elderly without a visit to the ED, and preventing future visits for those who do arrive. Paramedic Practitioner initiatives described above have been particularly valuable for elderly patients, whose minor conditionscan be treated in the home. A number of hospitals have specific protocols for streamlined management of “elderly fallers” which includes early evaluation in the Emergency Department or the ED’s Clinical Decision Unit by teams of staff including physiotherapists, occupational therapists and specialist nurses regarding their ability to be discharged back home. The University Hospital of Leicester established an Emergency Frailty Unit (EFU) where geriatricians perform a comprehensive assessment on elderly patients who are likely to be discharged within 24 hours. Additionally, care pathways were developed for the main ED and physicians in the EFU evaluated patients in that area as well. The assessments of the geriatricians can also be used for direct admissions to skilled nursing facilities, avoiding an inpatient admission. Preliminary data analysis showed that while the numbers of elderly patients attending the ED increased, the likelihood of admission was declined in comparison to historical controls. However, the relative risk for admission was also decreased in individuals under 64. 90-day readmissions among those over 85 were decreased by 13% [51].

Of course, England is not alone in recognising the growing impact of the aging populace. France has employed mobile geriatric teams for over a decade; as of 2008, there were 225 such units in the country [52]. The teams, consisting of a geriatrician, nurse and physiotherapist provide geriatric assessments for hospitalised patients and assist with care coordination until the patient is discharged. Moreover, in the ED, they provide assessment and advice for elderly patients. University Hospital, Grenoble has had such a unit since 1997 and is currently trialing the efficacy of home visits to prevent revisits to the hospital within a month of an emergency visit [53].

## Conclusion

New interventions in the delivery of health care have been tried in the UK and France to manage patient load on EDs. The evidence to date suggests that some of the interventions presented in this review, such as the establishment of admission assessment units and emergency care in the home, are very useful to manage patients with urgent care needs. On the other hand, interventions such as setting waiting time targets for EDs and telephone advice services may not be very effective.

Some of these interventions, such as emergency frailty units, provide more patient-centered care and also help to meet patients’ needs by providing a high quality service. The evidence has shown that alternative sites for urgent care improve patient access to healthcare services. Improving patients’ access is particularly important for urgent health problems. However, an unintended consequence of establishing alternative sites for urgent care and some telephone advice services appear to increase demand for medical services, rather than reducing ED visits Moreover, little is known about the cost-effectiveness of establishing alternative sites.

Therefore, an important consideration before replicating these interventions would be to know the primary purpose of implementing these interventions and to determine which service would be beneficial for that particular setting. In addition, the long-term impact of introducing these interventions on the emergency care system is also unknown. Thus, a sound evidence-based program evaluation needs to be developed in order to robustly evaluate new innovations.

## Abbreviations

NHS: National health service
GP: General practitioner
ED: Emergency department
